# Influence of Hydrogel and Zinc Oxide Nanoparticles on the Germination and Establishment of *Chenopodium quinoa*

**DOI:** 10.3390/life14091163

**Published:** 2024-09-13

**Authors:** José Luis Soto-Gonzales, José Vulfrano González-Fernández, Diego David Pinzón-Moreno, Eder Clidio Vicuña-Galindo, María Verónica Carranza-Oropeza

**Affiliations:** 1 Department of Chemical Engineering, Universidad Nacional Mayor de San Marcos, Lima 15081, Peru; diegodpinzon@gmail.com (D.D.P.-M.); evicunag@unmsm.edu.pe (E.C.V.-G.); mcarranzao@unmsm.edu.pe (M.V.C.-O.); 2 Instituto Tecnológico de San Luis Potosí, Tecnológico Nacional de México, San Luis Potosi 78437, Mexico; jose1.gf@slp.tecnm.mx; 3 Centro de Investigaciones Tecnológicas, Biomédicas y Medio Ambientales, Callao 07006, Peru

**Keywords:** hydrogel, quinoa, agricultural production, Andean grains, germination

## Abstract

The aim of this study was to assess the influence of hydrogel and zinc oxide nanoparticles on quinoa germination and establishment. Various doses of a commercial potassium-based hydrogel (0, 5, 7, and 9 g), each dissolved in one liter of rainwater, were applied. Additionally, 1.5 g of zinc oxide nanoparticles (ZnO-NP) and pre-crushed nitrogen fertilizer, at a rate of 1.6 kg/ha, were added to the solution to achieve a homogeneous mixture. Following the application of hydrogel in the 10-linear-meter rows corresponding to each treatment area in every block, 25 seeds per linear meter of the “Blanca de Juli” quinoa cultivar were sown with a 4 cm spacing between the seeds. Subsequently, a thin layer of soil, approximately 0.5 cm thick, was used to cover the seeds. Ten seedlings were randomly selected and labeled for subsequent evaluations. The experimental design employed in this research was a completely randomized block design. The collected data underwent an analysis of variance, and the means of all the treatments were compared using Tukey’s test with a 5% probability. Height and diameter evaluations of the plant neck were conducted every 45 days. The doses used in this study (5, 7, and 9 g of hydrogel per liter of water) significantly enhanced seed germination and increased the number of plants per linear meter (from 82.00 to 90.33) compared to the control dose without hydrogel (14.66), which resulted in an average of one plant per linear meter.

## 1. Introduction

Quinoa (*Chenopodium quinoa* Willd) is one of the most important agricultural crops in the Altiplano Region and is a part of food security [1]. Quinoa grains are sources of protein and essential amino acids and are higher in these components than other frequently consumed foods such as rice, barley, and wheat. Quinoa also possesses valuable proportions of bioactive compounds such as antioxidants and isoflavones [2].

Seed germination represents the initial physiological event in the phenology of cultivated plants. This process commences with the hydration of the seed. The germination process entails cellular and metabolic events, initiating with the absorption of water by dormant seeds and concluding with the elongation of the embryonic axis [3]. The scarcity of rainfall in the Peruvian Altiplano, where quinoa is produced, becomes a serious issue for agriculture and food security, mainly because there is no irrigation and dry soils with a low moisture content make it difficult for quinoa seeds to begin the germination process. In this regard, germination can be simplified into initial processes such as seed imbibition and metabolic activation, followed by seed coat rupture, and subsequent radicle emergence and seedling growth. The initial phase primarily involves water absorption, while the second phase is dependent on seed reserve mobilization [4]. In this context, an enhancement in the germination and biomass production of Trifolium, lettuce, and ryegrass in dune sand has been demonstrated in response to gel amendment compared to a control [5]. This indicates that the use of biodegradable hydro-retentive polymers can reduce the usage of natural resources such as fertilizers and pesticides [6].

Hydrogels are hydro-absorbent polymers, and according to their three-dimensional cross-linked structure and their absorption capacity, especially in their carboxylic groups, they can absorb 100 to 400 times their weight depending on the quality of water used [7]. Superabsorbent polymers can be used as a soil additive [8]. Hydrogels are products that are useful in irrigated and rainfed agriculture, precisely because of their capacity to retain and supply water to plants [9]. Studies have also been conducted to determine the effect of hydrogels on improving the growth and yield of crops such as beans and sugarcane [10]. The hydrogel generates a humid environment for the seeds and for the plants, which is why its use is recommended and why it has positive impacts on agriculture. The inclusion of water-retaining polymers is an option to facilitate the osmotic conditioning of seeds due to the fact that these polymers have the capacity to absorb large volumes of water [11].

NPs (nanoparticles) can be used in seed management [12]. Additionally, ZnO-NPs (zinc oxide nanoparticles) promote seed germination and seedling growth [13]. Zn, Cu, and Fe nanoparticles, either pure or mixed with silver, have been studied worldwide for their agricultural potential in plant growth promotion and for their use as nanofertilizers and antimicrobials, which significantly improve productivity. They are also efficient in reducing costs in the application of agrochemicals [14]. For lettuce, tobacco, and cotton, the rate of seedling emergence and the dry weight of seedlings increased in soil amended with a hydrogel [15].

An in vitro study demonstrated that zinc oxide nanoparticles (ZnO-NPs) obtained through green synthesis improved root and shoot growth, as well as biomass production, in *Juniperus procera* [16]. The use of nanoparticles (NPs) holds significant potential, as they can act as agents that promote cell division and growth, influencing the increase in enzymatic and antioxidant activity during the germination process [17]. The in vitro germination of Chenopodium quinoa seeds with ZnO nanoparticles resulted in significant changes in the germination rate among different treatments. Additionally, this treatment demonstrated a notable improvement in root development without any evidence of phytotoxicity [18].

One of the most common methods of estimating seed quality is the accelerated aging (AA) test, which can provide consistent information [19]. Its principle is that exposure to high temperatures and relative humidity has a significant impact on the rate of seed deterioration [20].

In this context, the objective of this work was to study the effect of hydrogel and Zn oxide nanoparticles on the germination and establishment of quinoa.

## 2. Materials and Methods

### 2.1. Location of the Experiment

The present investigation was carried out on a rural property intended for family agriculture in the department of Puno located in Jayllihuaya, with the following geographical coordinates: latitude 15°51′53.83″ S and longitude 69°59′15.09″ W.

### 2.2. Quinoa Seeds

The seeds were acquired in September 2020 from the National Institute of Agricultural Research (INIA), Salcedo Puno, Peru. The species used was *Chenopodium quinoa* of the Blanca de Juli cultivate, with a germination capacity of 96% according to the INIA [21]. The seeds originated from the 2019–2020 crop year.

### 2.3. Management and Installation of the Experiment

**Figure 1:**
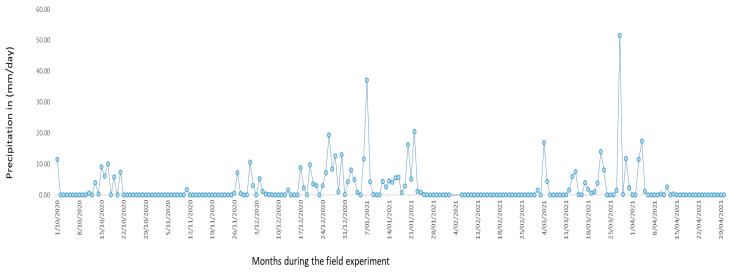
Months of rain in Puno during the experiment with quinoa in the field. Official data provided by the National Service of Meteorology and Hydrology SENAMHI, Peru.

The experiment was installed on 5 October 2021, the month in which farmers are accustomed to carrying out this activity in the Altiplano region. Between 26 November and 21 January, there were continuous rains, with the maximum rainfall being recorded on 7 January and 28 March 2021. Rain was important in this study (see Figure 1).

The experiment was installed in soil classified as Chincheros–Illpa (50–50%), from which 25 soil samples of 1 kg each were collected. These samples were placed to dry in the shade and then ground, mixed, and homogenized. Then, one sample was collected (considered a homogeneous sample), which was taken to the soil laboratory of the INIA Salcedo in Puno (see Table 1).

### 2.4. Base Fertilization

Based on the soil analysis, the fertilizer amount purchased from Yara International ASA (Oslo, Norway) was calculated for the fertilization of the *C. quinoa* crop, which required a formulation of NPK (80–40–00) for soil classified as Chincheros–Illpa (50–50%). Based on the soil analysis, it was not necessary to add phosphorus or potassium because the soil was rich in these nutrients. The area of the experiment corresponded to 20 × 7.20 m. The fertilizer calculation was 111.1 kg of urea per hectare (10,000 m^2^). The urea calculation was conducted to cover 144 m^2^, which corresponded to 1.6 kg of nitrogen fertilizer based on the total area of the experiment.

### 2.5. Hydrogels

Commercial polyacrylate hydrogels based on potassium (20–40 mesh, purity > 96%), branded as Plantagel (Lima, Peru), were used. The swelling capacity of the hydrogels was assessed in distilled water (pH ≈ 7) using the mesh bag method with a 200-mesh filter, according to the manufacturer’s instructions. Data were collected at various time intervals until the hydrogels reached a constant weight. The hydrogels underwent characterization through Fourier transform infrared spectroscopy–attenuated total reflectance (FTIR-ATR) utilizing a Shimadzu IRTracer-100 spectrometer (Kyoto, Japan). The analysis employed the Pike MIRacle single-reflection horizontal ATR device equipped with a ZnSe ATR crystal, operating in transmittance mode at 4500 cm^−1^. The experimental setup utilized 64 scans, and a resolution of 4 cm^−1^ was applied for the analysis.

### 2.6. Nanoparticles

The ZnO-NPs applied in this research were purchased from the US Research Nanomaterials Inc. company, located in Houston, TX, USA. The compound, quality, and elemental characteristics are indicated in Table 2 as reported by the manufacturer.

A morphological characterization of the ZnO-NPs was carried out by using transmission electron microscopy (TEM). The instrument was a Field Emission Gun, model Talos F200i, Thermo Fisher Scientific Inc. (Waltham, MA, USA) with a voltage of 200 kV and employing the bright-field mode. In order to characterize the stability of the ZnO-NPs in water, the zeta potential (zPot) method was used to measure the electrostatic potential of the ZnO-NPs. The instrument used to characterize the zPot was a NICOMP Nano Z3000 system, Particle Sizing Systems (Santa Barbara, CA, USA). A measure close to −30 mV (anionic) or 30 mV (cationic) was expected to avoid NP agglomeration and precipitation during the experimental procedures.

### 2.7. Determination of Zn Content in Samples

Samples of quinoa grains from each treatment, namely a dose of 5 g (D5g); a dose of 7 g (D7g); and a dose of 9 g (D9g), were taken to the chemical analysis laboratory at the Universidad Nacional Agraria La Molina (UNALM) and analyzed via atomic absorption spectrophotometry, using a PinAAcle 500 Flame AA Spectrometer (Shanghai, China).

### 2.8. Production

The production of quinoa grains was evaluated in tons per hectare (t ha^−1^) by scaling the experimental hydrogel doses (see Section 3).

### 2.9. Size of Root

The root size was evaluated for each plant in each treatment. To facilitate this, the plant was carefully extracted from the soil, and then the roots were washed and dried with a paper towel and subsequently stretched out on a table. The root size measurements are in cm (see Section 3).

### 2.10. Hydrogel Absorption of Water and Nutrients

Prior to the use of hydrogel in the field experiment, amounts of 5, 7, and 9 g in one liter of different types of water (rain, well, and tap water) were tested in order to observe the swelling and behavior of the hydrogel and, thus, decide as to which water would be best for the mechanism. The criteria were adapted using three replicates of each treatment, and once hydration was initiated, the amount of water retained was measured by draining the non-retained water and subtracting it from the initial amount. The hydration times were 20, 30, 60, 120, and 150 min [22]. The decision was made to use rainwater to ensure that the preparation was consistent and because it was the most appropriate in agriculture, and 60 min of swelling was chosen.

Three escalating doses of hydrogel, 5, 7, and 9 g, were hydrated in one liter of rainwater each, and 0 g of hydrogel was used as the control. The total water usage amounted to 480 L, distributed across 24 central furrows of the experiment at a speed of 5 km per hour. The hydrogel was measured in the following quantities: 5 g per liter of water, for a total of 100 g prepared in 20 L, distributed along 10 linear meters; 7 g of hydrogel per liter, totaling 140 g in 20 L, applied over 10 linear meters; and 9 g of hydrogel per liter, equivalent to 180 g in 20 L, applied along 10 linear meters of soil.

For each treatment, 1 L of water from the 20 L was taken, and 1.5 g of Zn oxide nanoparticles was dissolved for 1 min. Subsequently, this was mixed with the remaining 19 L of water. Crushed nitrogen fertilizer (urea powder), with the amount determined through a soil analysis, was then added at a ratio of 1.6 kg/ha, until a homogeneous mixture was achieved within the 20 L volume.

### 2.11. Quinoa Planting

Once the hydrogel was applied in the 10-linear-meter furrows, the seeds of *C. quinoa* Blanca de Juli cultivar were sown. They were distributed with a distance of 4 cm between seeds, totaling 25 seeds per 10 lineal meters, of which 10 plants were randomly selected for the experiment in each plot. After that, a covering was made with soil from the same plot to an approximate thickness of 0.5 cm using an iron rake.

### 2.12. Statistical Design

The statistical design used in this research corresponded to completely randomized blocks. The means of the treatments were processed with the statistical program ASSISTAT, version 7.7 beta [23]. The means were also discriminated using Tukey’s test with a 5% probability.

### 2.13. Evaluation of the Zn in Quinoa Seeds

For the study of the Zn in quinoa seed grains, 100 g was used for each treatment, and the evaluation was conducted at the end of the experiment using the atomic absorption method according to the manufacturer’s instructions.

### 2.14. Plant Height and Stem Diameter Evaluations

Evaluations of plant height (cm) and plant collar diameter (mm) were conducted every 45 days, with daily observations made throughout the study. In the field, no attempt was made to straighten the plants during height measurements, as all plants exhibited an erect growth habit.

### 2.15. Integrated Management of Pests and Diseases in Quinoa Plants 

Pest control was conducted using yellow sticky traps made with plastic bags impregnated with common motor oil to control moths, mainly according to the suggestions in [24]. Bags with nets were also used to protect the plants in the phenological phase of physiological maturity against attacks by birds, mainly pigeons (*Columba livia*), which are present in the last phenological phase of the quinoa crop. Two types of commercial fungal antagonists of the genus Trichoderma (*Trichoderma harzianum* and *Trichoderma viride*) from the brand PBA Productos Biológicos Para Agricultura Perú (Lima, Peru), were also used as a preventive measure for the control of quinoa “mildew”, as recommended in [25]. For the control of the quinoa moth, pheromone traps containing pheromones for “ticonas” of quinoa, *Agrotis ipsilum*, from the brand Pherobank B.V. (Utrecht, The Netherlands), were used.

### 2.16. Accelerated Aging Test on Chenopodium Quinoa Seeds

Gerbox boxes [26] were used, containing a tray with a millimeter plastic mesh where the seeds were distributed to form a uniform layer, preventing contact with the water inside the gerbox. Inside each gerbox, 45 mL of distilled water was added; the gerbox boxes were kept in a germinator chamber at 40 and 45 °C for 48 h. Subsequently, the seeds were subjected to a germination test before and after the AA test. The water content of the seeds was determined before and after the AA test in a sample of seeds that entered the germinator chamber; afterwards, the aged seeds were allowed to germinate. Before and after the AA test, the water content of the seeds was determined after drying in an oven at 105 ± 3 °C for 24 h, using two subsamples of 1000 mg of seeds, as suggested by the Rules for Seed Analysis [27].

### 2.17. Statistical Design to Evaluate the Accelerated Aging Test on Chenopodium Quinoa Seeds

The experiment was conducted using a randomized block design. Treatments with a composite lot and four replications of 25 seeds were used. The means obtained from the analysis of variance for each variable were compared using Tukey’s statistical test with a 5% probability.

## 3. Results

In this research, we chose to use rainwater to swell the hydrogel. Since the properties of water influence the swelling of the hydrogel, a chemical analysis of the rainwater was conducted, where the main parameters obtained were a pH of 7.55, turbidity of 0.98 NTU, and electrical conductivity of 13.51 µS/cm (Table 3).

### 3.1. Hydrogel Characterization

**Figure 2:**
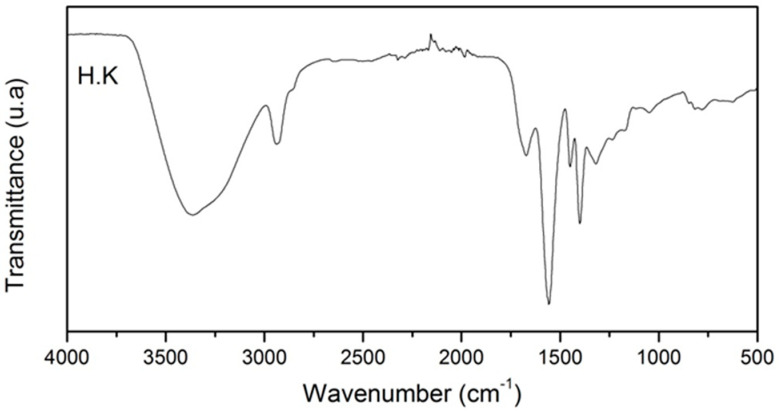
FTIR spectrum of the hydrogel.

**Figure 3:**
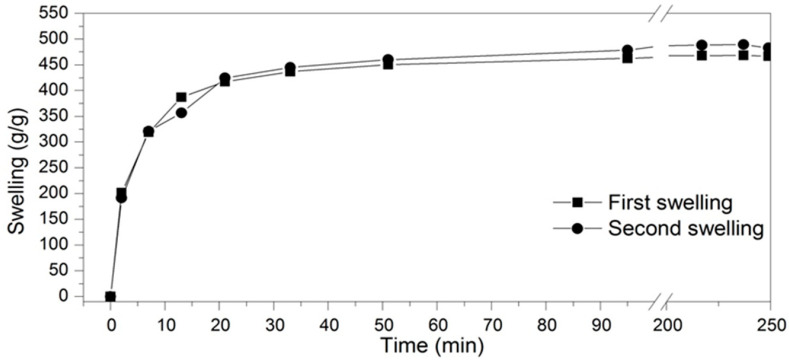
Swelling kinetics of the hydrogel.

A commercial polyacrylate hydrogel based on potassium (20–40 mesh, purity > 96%) was purchased, with the characteristic FTIR bands recorded in Table 4 and Figure 2. Figure 3 illustrates the first and second kinetic swelling curves of the commercial potassium-based hydrogel when exposed to water. It was noted that, generally, the potassium-based hydrogel demonstrated a swelling ratio at equilibrium of approximately 475 g/g. Similarly, the hydrogel reached 90% of their swelling capacity in about 20 min.

### 3.2. Nanoparticle Characterization

In this research, ZnO-NPs were used as a growth promoter [8]. The provided material acted as nutrients in the experiment and consisted of a commercial ZnO-NP powder with a high purity and a diameter of 35 to 45 nm. The nanopowdered material was dissolved in water and mixed with urea for application in the field.

**Figure 4:**
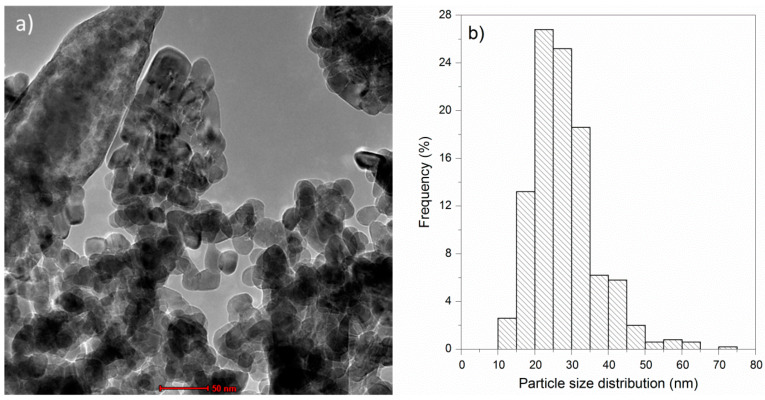
(**a**) TEM micrograph of ZnO nanoparticles and (**b**) histogram of particle size distribution. Scale bar in (**a**) is 50 nm.

**Figure 5:**
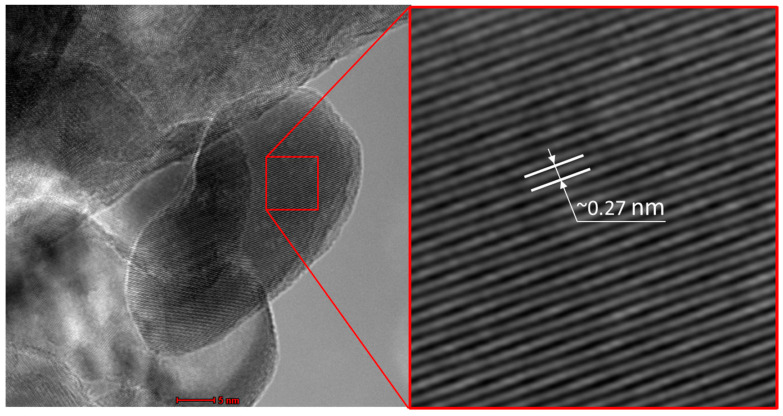
High-resolution TEM micrograph of ZnO nanoparticles. Scale bar in (**left**) is 5 nm.

Figure 4a shows a TEM micrograph of the ZnO nanoparticles used in the experiments. These nanoparticles exhibited a rounded morphology with irregular shapes. On the other hand, Figure 4b presents a histogram showing the particle size distribution in terms of frequency. The histogram revealed an average size of approximately 28 nm. Additionally, based on the obtained data (500 measurements), particles with minimum and maximum sizes of 10.8 nm and 72.1 nm, respectively, were observed. Figure 5 presents a high-resolution TEM micrograph showing the grain growth orientation along the c axis. Additionally, it was possible to measure the interplanar distance corresponding to the (002) plane, which was approximately 0.27 nm.

**Figure 6:**
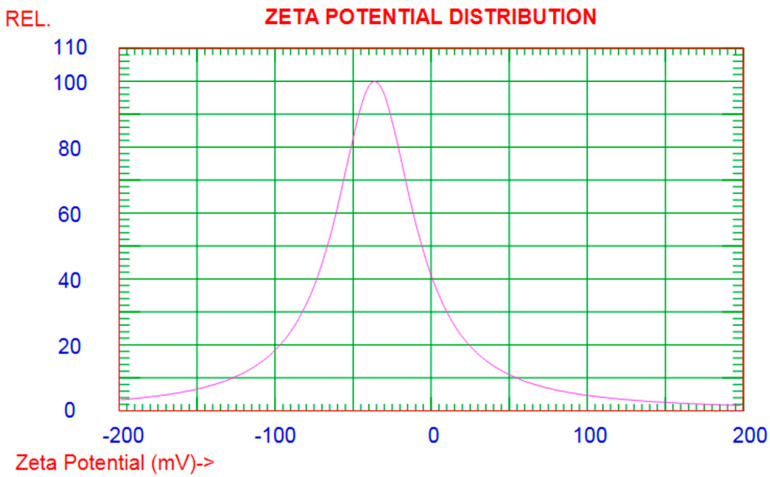
Zeta potential distribution of commercial ZnO-NPs.

As previously discussed, the zeta potential magnitude served as an indicator of the potential stability of the ZnO-NPs in an aqueous medium. In the case of the commercial ZnO-NP sample, the zPot value measured was −36.26 mV at room temperature (refer to Figure 6), suggesting a high level of stability for the particles. It is important to note that the term ‘stability’ in this context refers specifically to the resistance of nanoparticles to agglomeration rather than to chemical degradation. Agglomeration, which is the clumping together of nanoparticles, can be driven by forces such as solvation, van der Waals forces, electrostatic attractions, and hydrophobic interactions. Zeta potential measurements exceeding +/−30 mV indicate that the electrostatic repulsion between particles is sufficient to prevent such agglomeration, thereby maintaining a stable dispersion of nanoparticles in the solution.

However, stability in a broader sense could also refer to the chemical stability of the nanoparticles, meaning their resistance to degradation or changes in their chemical structure over time. Degradation involves the breakdown of nanoparticles into smaller components or the alteration of their surface chemistry, which can lead to a loss of functionality. In this study, while zeta potential is used to assess stability against agglomeration, it does not provide direct information about the chemical stability or degradation of the nanoparticles. For a complete assessment of stability, both aspects—agglomeration and degradation—should be considered.

### 3.3. Hydrogel Application and Statistics

Table 5 and Table 6 show all the variables studied based on increasing doses of urea and ZnO loaded in the hydrogel. There were significant differences (*p* < 0.01, *p* < 0.05) for the different growth stages in quinoa plants.

After the quinoa crop finished its phenological stages 180 days after harvest, the results showed significant differences for *Chenopodium quinoa* in terms of the parameters presented in Table 5, where the maximum growth in plant height was achieved with the doses of 5 and 7 g of hydrogel. In the present investigation, the doses of 5 and 7 g of hydrogel provided a larger plant size. This effect was also found when the size of the plants was analyzed, as shown in Table 5, and can probably be attributed to the water availability in the soil due to the action of the hydrogel.

In this study, the ZnO-NPs and urea used in the solution, together with the hydrogel, helped activate the embryos of the seeds and demonstrated encouraging effects throughout the crop cycle. Negative effects were observed for the plant height from days 45 to 180 in the control treatment without hydrogel, with a lower mean (Table 5) when compared to the other treatments that did have the commercial hydrogel. The control treatment also obtained a low percentage of germination, with an average of 6.49, and plant production per linear meter, with an average of 14.66. The other hydrogel treatments surpassed the control in seed germination, with averages varying from 45.75 to 50.99, and in the number of plants per linear meter, with means varying from 82.00 to 90.33 (see Table 6).

In Table 5, the coefficients of variation for plant height ranged from 4.78 to 11.94. The growth of the quinoa plants may have been affected by genetic factors, as these plants have undergone some degree of genetic improvement by the INIA in Peru over time. This was reflected in the almost uniform growth and field performance of the plants in question. A significant difference was found for the effect of doses on the plant height and plant diameter (*p* < 0.05; see Table 5). The doses (5, 7, and 9 g of hydrogel per liter of water) used in this research promoted the growth in height and diameter of quinoa plants compared to the treatment of 0 g of hydrogel and without ZnO-NPs (control treatment), for which the growth was lower. There were no significant differences between treatments 7 and 9, which in turn resulted in larger plant sizes when compared to the 5 g dose at the evaluation after 45 days. The hydrogel hydrated with water and mixed with nutrients (Zn and N) in all doses absorbed enough water to activate the embryos of the seeds that germinated in this substrate. 

The germination percentage in the hydrogel treatments surpassed that in the control treatment (D0g). This distinction is notable, particularly considering the absence of rainfall during these days. The quinoa seeds, influenced by the available humidity, initiated the germination process. For a visual representation, refer to Figure 1, which illustrates the precipitation levels during the period when the Chenopodium quinoa crop was present in the field in Puno. During the first week (from 02 to 09/10/20), when the quinoa seeds were sown, no rain was recorded (Figure 1), except on 01/10/21, when water was collected by the plants for hydration from the hydrogel.

For the Zn in the quinoa grain samples, there were no significant differences among the treatments. Regarding the leaf area, the 7 g dose resulted in a higher percentage in this evaluated characteristic. Regarding the grams of yield per plant (Table 6), there were no significant differences among treatments, with the highest production of quinoa being 1.874 (D7g) t/ha. For the root size, there were no significant differences, probably because the roots were pulled out of the soil. The soil loses water by evaporation, leaving the roots brittle. The remains of the roots were likely left in the soil; in addition, during that period (April) of harvest, no rainfall was recorded (Figure 1), which made it difficult to extract the quinoa roots.

This research revealed that the control dose of 0 g (without the use of hydrogel) resulted in an extremely low germination rate of 6.49, due to the scarcity of moisture in the soil and the total lack of rainfall in the week that the seeds were placed in the soil. In comparison, the doses of 5, 7, and 9 g of hydrogel resulted in a good percentage of germinated seeds, which was reflected in the high percentage of adult plants per linear meter.

Among the 25 seeds spaced 4 cm apart in the control treatment (D0g), only 10 germinated, and these individuals survived until the conclusion of the experiment. Notably, an experimental plot with more space for growth was observed. In contrast, the other three treatments (D5g, D7g, and D9g) contained all 25 plants in good phytosanitary condition, with only 10 samples studied.

Quinoa plants (refer to Table 6) were distributed with an approximately 10 cm gap between them in the treatments (D5g, D7g, and D9g) due to the sowing process. The application of hydrogel played a crucial role in ensuring that this plant distribution allowed for their sustained vitality and parallel growth, fostering healthy competition for space and nutrients. This contrasted with the control treatment (D0g), where several quinoa plants failed to germinate and maintain the 10 cm gap between them in the field.

The treatment of 0 g of hydrogel resulted in a low number of plants per linear meter (see Table 6); the results indicated that there was only one plant per linear meter on average. Under normal conditions and without the use of hydrogel, we would have problems in terms of deficient planting per m^2^ and per hectare, leading us to carry out a second sowing (replanting), which would be subject to input expenses such as seeds, machinery, logistics, and all the technical itineraries for the cultivation of quinoa.

The hydrated hydrogel used in this research generated a high germination rate in the treatments. A good number of quinoa plants per linear meter was found, precisely because the sowing was carried out during a period of low rainfall (see Figure 1) and according to the crop calendar for the Puno region.

**Figure 7:**
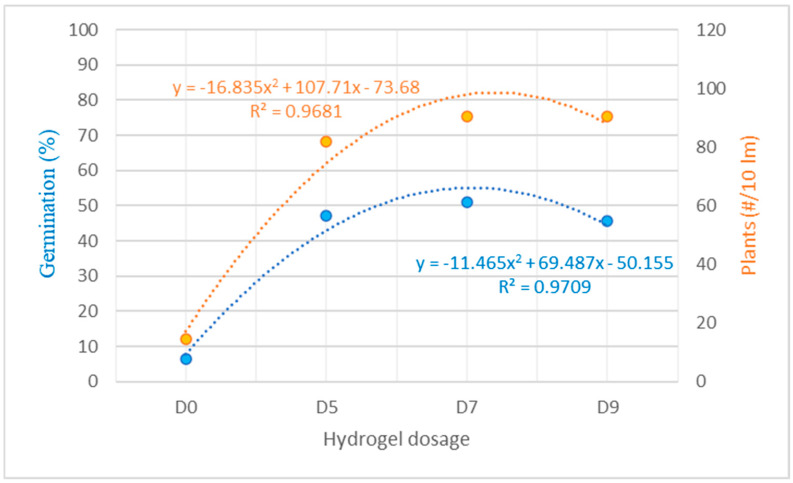
Regression analysis for the germination percentage and the number of plants from a population of 25 seedlings in 10 linear meters of sowing. D0, D5, D7, and D9 correspond to the different doses of hydrogel and ZnO-NPs in the Chenopodium quinoa—Blanca de Juli.

Figure 7 shows the regression analysis for the germination percentage (seedling emergence through the soil) and the number of plants from a population of 25 seedlings in 10 linear meters of sowing. 

In terms of the germination percentage and plant count, the control treatment without hydrogel and ZnO-NPs (D0) resulted in the lowest percentages of germinated seeds (6.49) and seedlings (14.66). This outcome was attributed to the absence of hydrated hydrogel and ZnO-NPs in the soil, coupled with the lack of rainfall during sowing. These values were statistically significantly lower than those for treatments involving hydrogel with ZnO-NPs. Notably, doses D7 and D9 resulted in a high performance in moisture retention efficiency, stability, and ZnO-NP adherence to the seeds. Under field conditions, this resulted in a germination percentage of 50.99 for D7 and a number of plants of 90.33, while the germination percentage for D9 was 45.73. For the number of plants, D7 and D9 yielded the best means, resulting in equivalent values (90.33 plants). This indicates that the use of hydrogel at these doses promoted the growth of a significant number of plants per 10 linear meters in this study.

Table 7 shows the data from the analysis of variance in an experiment applying accelerated aging on the quinoa seeds used in the present experiment, where the two treatments were not significant. The accelerated aging test conducted at two different temperatures destroyed the seeds at both 40 and 45 °C, resulting in low germination percentages and effects on the other studied parameters, such as the daily average germination and the germination speed index. In Table 6, the treatment with the 0 dose of hydrogel showed an extremely low germination percentage of 6.49%. Meanwhile, the germination rates of the other treatments with ZnO-NPs were significantly higher, varying from 47.09 to 50.99%. This leads us to believe that the zinc nanoparticles effectively aided in germination (Table 6). In the present study (Table 7), seeds subjected to accelerated aging had germination percentages ranging from 4% to 1.75%, which is extremely low. 

## 4. Discussion

Plant height is a good parameter to consider in phytotechnology; it is an indicator of plant quality, since the height of a plant is related to plant vigor [28]. It was verified that the use of 6 g of hydrogel in the substrate provided satisfactory results for the evaluated parameters of the height and neck diameter of *Anadenanthera peregrina* plants [29].

The hydrogel also increased the total porosity, without affecting the water availability, and provided an increase in the volume of reserve water in the substrate, favoring the performance of the aerial part of the plants [30]. The use of hydrogel loaded with ZnO-NPs is necessary in quinoa production in the highlands, given that water resources are scarce, and rainfall is irregular, probably due to climate change.

In a study employing ZnO-NPs on sorghum *Pennisetum americanum*, significant increases in the stem growth, root length, root area, and other characteristics were observed compared to a control treatment [31]. Additionally, other studies have indicated that zinc oxide (ZnO), silver (Ag), and titanium dioxide (TiO_2_) nanoparticles enhance the germination speed in peanut seeds [32], which is consistent with our results for quinoa, as can be observed in Table 5 for plant height.

In this study, the statistical analysis revealed a low coefficient of variation (Table 5 and Table 6), indicating the favorable condition of low coefficients for an agricultural species [33]. The quantity of water needed for hydration increased with a greater amount of hydrogel in the soil [34]. Different doses of hydrogel were deliberately employed in this study to explore this aspect. Upon hydration, the polymer gradually releases water into the soil [35]. Notably, the utilization of hydrogel eliminates the need for a specific soil moisture content [36], which would otherwise supply moisture to the seeds and initiate physiological processes within the embryos.

Water is fundamental for cell metabolism during germination, playing a crucial role in enzymatic activity, solubilization, and the transport of photo-assimilates. It acts as a facilitator in the digestion of seed reserves [37]. Supporting the hypothesis for hydrogel treatments, a higher seed germination was observed.

Also, Manaila et al., 2023 developed a new hydrogel based on sodium alginate–g-acrylic acid. Their study found that the hydrogel swelled consistently in response to rainwater, considering that rain is the most commonly used source of water in agricultural fields [38]. Based on these data, rainwater was used in this research.

On the other hand, in a study of spring and well waters in Puno, Peru, Brousett et al., 2018 showed that the pH in springs ranged from 7.44 to 7.12, and for well water, the pH ranged from 8.38 to 6.74. The turbidity was also studied, with spring water ranging from 2.66 to 2.12 NTU and well water from 0.98 to 1.11 NTU. The electrical conductivity was very high in spring water, ranging from 277 to 215 µS/cm, and in well water, it ranged from 221 to 292 µS/cm [39].

Studies conducted by several researchers that have applied hydrogel for various crops, including *Eucalyptus dunnii* [40], *Corymbia citriodora* [41], and *Avena sativa* [42], have indicated that hydrogel has a positive influence on plant growth, as it promotes the availability of nutrients and water. In addition, ZnO-NPs support plant growth and metabolism, and, as a consequence, plant health, for cauliflowers and tomatoes [43]. Regarding the utilization of ZnO-NPs in the present study, it is plausible that materials with nanometer dimensions and larger surface areas could enhance the absorption, translocation, and retention of nutrients in plant species [44]. However, in this research, the impact of ZnO-NPs on these terms and variables mentioned requires further investigation, where the influence of these nanomaterials on crops is methodologically specified.

In this investigation, a good yield was obtained with the applied treatments. The literature indicates that it is possible to obtain average yields of 5 t/ha [45]. Under the current conditions, the average yield for Puno is 1.1 t/ha [46]. Records report reaching 1497 kg per hectare with the cultivation of White Juli Quinoa for the 2015–2016 agricultural season in the department of Puno, Peru [47].

For the hydrogel treatments, there was a high density of plants in the soil (Table 6). In soil research with string beans in large plant populations, the existence of a direct effect on the productive capacity of these plants was indicated with a decrease in the number of inflorescences and flowers per plant [48,49]. Ideal plant densities directly influence the yield and productivity components [50]. Also, the wider spacing of approximately 80 cm between plants in the control treatment facilitated unrestricted development without competition for space and nutrients. It is plausible that the treatments (D5g, D7g, and D9g), with a large population of Quinoa plants, experienced this effect of competition, contrary to the treatment with zero grams of hydrogel.

The results obtained from the accelerated aging test showed that quinoa seeds subjected to temperatures of 40 and 45 °C experienced a significant reduction in their germination percentage, with extremely low values (4% to 1.75%). This suggests that high temperatures negatively impact seed viability. Parameters such as the MDG and GSI did not show significant differences among the treatments, suggesting that the main impact of ZnO nanoparticles was observed on the initial germination rate. Overall, the results suggest that the use of ZnO-NPs can be beneficial for improving germination under thermal stress conditions.

## 5. Conclusions

The various doses applied in this study—5, 7, and 9 g of hydrogel per liter of water, along with Zn oxide nanoparticles—significantly promoted seed germination, enhanced growth, and increased the number of plants per linear meter. The averages ranged from 82.00 to 90.33, presenting a notable contrast to the control dose without hydrogel (14.66), where practically only one plant per linear meter was obtained. The doses of 5 and 7 g of hydrogel promoted maximum growth in the treatments, with means ranging from 129.33 to 139.56 cm, respectively. The use of hydrogel in the cultivation of quinoa can be useful for germination by accelerating the physiological processes of the embryo during periods of low rainfall, as observed in this study. The absence of hydrogel in the control treatment (0 g dose) resulted in an exceptionally low mean germination percentage of 6.49%, in stark contrast to the 50.99% observed with a dose of 7 g per liter of water. This significant difference underscores the efficacy of a hydrogel application. Therefore, based on these compelling results, the utilization of hydrogel is strongly recommended for quinoa production in Puno.

## Figures and Tables

**Table 1:** Fertility and salinity analysis of a soil sample from Jayllihuaya Puno, Peru.

	C.E.					Mechanical Analysis			Class	CIC	Exchangeable Cations					Sum	Sum	%
pH	(1:1)	CaCO_3_	M.O.	P	K	Sand	Slit	Clay	Texture		Ca^+2^	Mg^+2^	K^+^	Na^+^	Al^+3^ + H^+^	of	of	Sat. of
(1:1)	dS/m	%	%	ppm	ppm	%	%	%			meq/100 g					Cations	Basis	Basis
7.91	2.71	0.00	2.89	10.68	606.01	41	48	11	F	14.80	7.30	3.40	1.10	1.50	0.00	13.30	13.30	90

**Table 2:** Characteristics of the ZnO-NPs used in this research.

ZnO	Cu	Mn	Cd	Pb
>99%	<3 ppm	<5 ppm	<9 ppm	<9 ppm
Zinc oxide nanopowder (ZnO)				
Nanoparticle (ZnO) purity: 99+%				
Nanoparticle (ZnO) APS: 35–45 nm				
Nanoparticle (ZnO) SSA: ~65 m^2^/g				
Nanoparticle (ZnO) color: milky white				
Nanoparticle (ZnO) crystal phase: single crystal				
Nanoparticle (ZnO) morphology: nearly spherical				
Nanoparticle (ZnO) true density: 5.606 g/cm^3^				
ZnO-NP certificate of analysis was provided by US Research Nanomaterials Inc. (https://www.us-nano.com/inc/sdetail/354, accessed on 28 November 2023).				

**Table 3:** Chemical analysis of rainwater used in polymer hydration in Jayllihuaya, Puno, Peru.

pH	Conductivity	Sulfate	Nitrite	Nitrate	Chlorides	Turbidity	Total Hardness	Total Dissolved Solids
	µS/cm	mg SO_4_^−2^/L	mg NO_2_^−^/L	mg N-NO_3_^−^/L	mg Cl^−^/L	NTU	mg CaCO_3_/L	mg/L
7.55	13.51	2.13	<0.01	<0.20	1.49	0.98	5.96	36.67

**Table 4:** Characteristic bands of the hydrogel based on potassium.

	Description	Deformation	Wavenumber (cm^−1^)
Hydrogel based on potassium polyacrylate	Stretching vibration of the hydroxyl group.	O-H	~3369
	Asymmetric and symmetric stretch.	CH_2_	~2935 and ~2860
	Deformation vibrations.	C-OH	~1674
	Asymmetric and symmetric stretching and another associated deformation of the group.	COO^−^	~1555, ~1451, ~1404, ~1317, and ~1169
	Stretching vibrations of the C-O bond and deformation vibrations of the C-O-H group.	C-O and C-O-H	~1239
	Bond deformation.	C–C	1162
	Bond stretching in the carboxyl acid structure.	C=O	~1113
	Other characteristic deformations of the polymeric hydrogels based on potassium.	--	~855, ~820, ~784, ~638, and ~616

**Table 5:** Analysis of variance in plant height and collar diameter at 45, 90, 135, and 180 days after planting, at different doses of hydrogel and ZnO-NPs in the *Chenopodium quinoa* Blanca de Juli cultivar.

Sources of Variation	Mean Squares							
	Plant Height (cm)				Plant Diameter (cm)			
	Days 45	90	135	180	45	90	135	180
F treatments	199.10 **	25.74 **	0.54 ^ns^	8.36 *	22.93 **	69.33 **	3.17 ^ns^	32.37 **
Error	1.87	36.33	48.86	90.06	0.60	1.50	22.38	41.01
CV (%)	4.78	8.19	10.15	8.43	10.62	6.64	11.94	7.5
LSD	1.472	5.960	22.857	29.544	0.815	1.022	5.188	5.688
Means								
D0g	4.80 ^c^	16.86 ^b^	75.90 ^a^	98.93 ^b^	1.58 ^b^	3.18 ^c^	13.65 ^a^	18.73 ^c^
D5g	11.19 ^b^	28.58 ^a^	83.96 ^a^	129.33 ^a^	2.80 ^a^	5.02 ^b^	15.93 ^a^	24.48 ^b^
D7g	13.33 ^a^	30.90 ^a^	80.43 ^a^	139.56 ^a^	3.02 ^a^	6.46 ^a^	17.76 ^a^	30.29 ^a^
D9g	14.16 ^a^	26.56 ^a^	78.16 ^a^	127.66 ^ab^	3.44 ^a^	7.09 ^a^	14.06 ^a^	33.74 ^a^

Means followed by the same letter, in each column, for each time of evaluation, are statistically different (Tukey; *p* < 0.05). F: variance ratio. CV: coefficient of variation (%). LSD: least significant difference. ns = not significant (*p* > 0.05). * = *p* < 0.05. ** = *p* < 0.01.

**Table 6:** Analysis of variance for Zn in quinoa grains, leaf area, yield in grams per plant and tons per hectare, root size in cm at 180 days after planting, % germination, and number of plants per linear meter at different doses of hydrogel and ZnO-NPs in the Chenopodium quinoa Blanca de Juli cultivar.

Sources of	Zn	Leaf Area	Yield	Size of	% of	Number of
Variation	(mg)	(cm^2^)	gr/pl and t/ha	Root (cm)	Germination	Plants per Linear Meter
F treatments	0.60 ^ns^	10.55 **	0.5872 ^ns^	1.50 ^ns^	263.80 **	539.449 **
Error	31.33	5.89	61.10	10.32	89.47	124.00
CV (%)	6.72	3.75	28.65	7.49	5.92	3.94
LSD	5.161	87.858	28.010	3.454	5.813	7.733
Dose						
D0g	---	765.33 ^b^	35.13 ^a^ (1.756)	16.167 ^a^	6.49 ^b^	14.66 ^c^
D5g	26.11 ^a^	821.66 ^ab^	37.40 ^a^ (1.870)	15.167 ^a^	47.09 ^a^	82.00 ^b^
D7g	26.890 ^a^	906.33 ^a^	37.49 ^a^ (1.874)	17.167 ^a^	50.99 ^a^	90.33 ^a^
D9g	27.997 ^a^	819.00 ^ab^	28.19 ^a^ (1.409)	16.733 ^a^	45.73 ^a^	90.33 ^a^

Means followed by the same letter, in each column, for each time of evaluation, are statistically different (Tukey; *p* < 0.05). F: variance ratio. CV: coefficient of variation (%). LSD: least significant difference. ns = not significant (*p* > 0.05). ** = *p* < 0.01.

**Table 7:** Analysis of variance for accelerated aging at 40 and 45 °C, germination percentage, mean daily germination, and germination speed index.

Sources of	Mean Squares		
Variation			
	% G	MDG	GSI
F	9.00 *	1.76 ^ns^	0.17 ^ns^
Mean squares	10.12	1.16	0.00004
LSD	1.83	1.39	0.02
CV	36.98	11.29	96.19
Accelerated aging			
AA 40 °C	4.00 ^a^	6.73 ^a^	0.017 ^a^
AA 45 °C	1.75 ^b^	7.50 ^a^	0.013 ^a^

Means followed by the same letter, in each column, for each time of evaluation, are statistically different (Tukey; *p* < 0.05). F: variance ratio. CV: coefficient of variation (%). LSD: least significant difference. ns: not significant (*p* > 0.05). * *p* < 0.05. G: germination percentage. MDG: mean daily germination. GSI: germination speed index.

## Data Availability

Dataset available on request from the authors.
